# Bioactive Compounds and Antioxidant Capacity in Anthocyanin-Rich Carrots: A Comparison between the Black Carrot and the Apulian Landrace “Polignano” Carrot

**DOI:** 10.3390/plants10030564

**Published:** 2021-03-17

**Authors:** Federica Blando, Stefano Marchello, Gabriele Maiorano, Miriana Durante, Angelo Signore, Maura N. Laus, Mario Soccio, Giovanni Mita

**Affiliations:** 1 Institute of Sciences of Food Production, CNR, Via Prov.le Lecce-Monteroni, 73100 Lecce, Italy; stefano.marchello88@gmail.com (S.M.); gabriele.maiorano@ispa.cnr.it (G.M.); miriana.durante@ispa.cnr.it (M.D.); giovanni.mita@ispa.cnr.it (G.M.); 2 Department of Agricultural and Environmental Science, University of Bari Aldo Moro, Via Amendola 165/A, 70126 Bari, Italy; angelo.signore@uniba.it; 3 Department of Agriculture, Food, Natural Resources and Engineering, University of Foggia, 71100 Foggia, Italy; maura.laus@unifg.it (M.N.L.); mario.soccio@unifg.it (M.S.)

**Keywords:** acylated anthocyanin, purple carrot, polyphenolic compounds, carotenoids, antioxidant activity, organic acid, fructose, glucose, vegetable biodiversity

## Abstract

The carrot is one of the most cultivated vegetables in the world. Black or purple carrots contain acylated anthocyanins which are of special interest to the food industry for their stability and nutraceutical characteristics. Anthocyanin-rich fruits and vegetables have gained popularity in the last ten years, due to the health benefits they provide. In this paper, the characterizations of the bioactive compounds and antioxidant capacities of different anthocyanin-containing carrots (a black carrot—BC, and a local purple carrot, the “Polignano” carrot—PC), compared to the commercial orange carrot (OC) (lacking of anthocyanins), are reported. The anthocyanin profiles of the polyphenolic extracts of BC and PC were similar, but differences were observed at quantitative levels. The total anthocyanin content in BC was more than twice that in PC (13.84 ± 0.61 vs. 5.64 ± 0.48 mg K Eq. g^−1^ DW). Phenolic acids (mostly chlorogenic acid) were also present at high level in anthocyanin-rich carrots compared to OC. High polyphenol content accounted also for a high reducing capacity (evaluated by Folin–Ciocalteu reagent, FCR), and antioxidant capacity (evaluated by TEAC and ORAC assays) which were the highest for BC (FCR value: 16.6 ± 1.1 mg GAE. g^−1^ DW; TEAC: 76.6 ± 10.6 µmol TE. g^−1^ DW; ORAC: 159.9 ± 3.3 µmol TE. g^−1^ DW). All carrot genotypes (mostly OC) were rich in carotenoids (BC 0.14 ± 0.024; PC 0.33 ± 0.038; OC 1.29 ± 0.09 mg. g^−1^ DW), with predominance of α and β-carotene, in OC, and lutein in BC. PC showed the highest malic acid and sugar (glucose plus fructose) content. In conclusion, while BC is remarkable for nutraceutical features, the local genotype (“Polignano” carrot) is worth considering in genetic biodiversity conservation programme.

## 1. Introduction

The carrot (*Daucus carota* L.) is a biennial herbaceous species, belonging to the Apiaceae family. Among vegetables, carrots are one of the most cultivated, with a worldwide production of around 45 million tonnes, half of which is accomplished in China [1]. They are also being increasingly consumed, thanks to their ability to keep for a long time, pleasant flavour/taste and perceived health benefits related to their nutritional value. This last aspect has made carrots more popular with consumers and more widely cultivated in the world. With a total carotenoid content up to 55 mg/100 g, the carrot is one of the best vegetable sources of carotenes [2]; it provides a significant amount of dietary vitamin A, through its precursors (α and β-carotene). Moreover, other vitamins (ascorbic acid, thiamine, riboflavin and niacin), minerals (high potassium content) and dietary fibre are found in carrots [3]. In addition to those nutrients, black carrots contain anthocyanins, which increase their nutritional value.

The black or purple carrot (*Daucus carota* ssp. *sativus* var. *atrorubens* Alef.) belongs to the Eastern group of the originally domesticated carrots, having its primary domestication centre today in the Kashmir-Afghanistan-Turkestan regions [4], as has been reported by molecular data as well [5]. The Western group, with carotenoid-pigmented roots, evolved thanks to the Eastern group, and by the 17th century took the place of the purple types in Europe, through human preference and selection [4].

Given the growing interest in multi-coloured carrots, some purple carrot cultivars have been released over the last few years [6]. However, as carrot breeding programmes move forward, the expansion of carrot global markets (including black carrot) and more aware consumers will push the research toward this direction [7]. In fact, the black carrot is among the most used anthocyanin sources as a food colourant, due to its high stability to the processing conditions and storage [8,9,10]. Moreover, black carrot extract shows intense antiradical and antioxidant activities [11].

Anthocyanins are a sub-class of polyphenols, naturally occurring pigments responsible for the red to dark blue colour of flowers, fruits and vegetables, and they have been studied extensively in the last few years because of their health promoting properties as antioxidant compounds [11,12]. Moreover, several health benefits have been ascribed to anthocyanins, not only related to their antioxidant activities. In vivo studies on humans and model animals have reported potential anti-diabetic, anti-inflammatory and cardioprotective activities, along with hepato- and nephroprotection, and anti-atherogenic activities, among others [13,14].

Due to that scientific evidence, the consumption of fruits and vegetables containing anthocyanins has been fostered also by the public health services of the Western countries [15]. As a consequence, consumers have been made aware of the health promoting properties of anthocyanin-rich foods, and manufacturers have shown a growing interest in including those bioactive compounds in processed foods (juice, candies, confectionary, ice cream, etc.) as a healthier alternative to synthetic colourants (FD&C Red40 or E129) [16].

Anthocyanin-containing carrots have been developed inside modern carrot breeding programmes, to increase the nutritional quality and visual appearance of the food supply [17,18]. On the other hand, in Italy (Apulia Region), local ecotypes, such as the “Polignano” carrot, have been discovered and valorised through specific actions under the “Rural Development Programme” aimed at preserving and protecting regional genetic biodiversity [19,20,21], or valorising such local varieties by the means of biofortification with iodine [22]. It is therefore important to study the phytochemical characteristics of these local genotypes, in order to valorise them and to avoid genetic erosion, and because it is known that local genotypes are best suited for local environments [23].

The aim of this study was to characterize the phytochemical profiles of different anthocyanin-containing carrots (a black carrot and a local purple carrot, the “Polignano” carrot), compared to the commercial orange carrot, with a special emphasis on the antioxidant components.

## 2. Results and Discussion

The black carrot has been extensively studied for its nutraceutical characteristics that impart outstanding health properties [24]. Moreover, it has been studied in-depth for processing, in order to extend its availability all over the year, as it is a perishable vegetable [10]. The phytochemical composition, particularly in relation to anthocyanins, has been reported in detail [8,25,26,27]. At the molecular level, the biosynthetic pathways leading to anthocyanins have been also studied in the black carrot [7,28].

Our study was undertaken with the aim of giving an overview of the black carrot (BC) and a local landrace of purple carrot, the “Polignano” carrot (PC) from a nutraceutical point of view, compared to the commercial orange carrot (OC).

**Figure 1:**
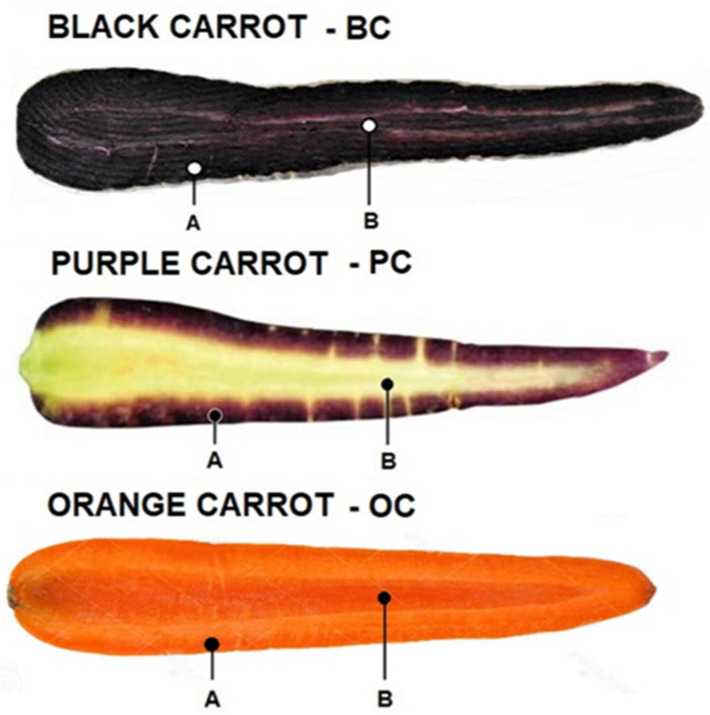
Longitudinal sections of differently coloured carrots. A and B indicate the phloem (cortex) and xylem (core) tissues, respectively.

**Figure 2:**
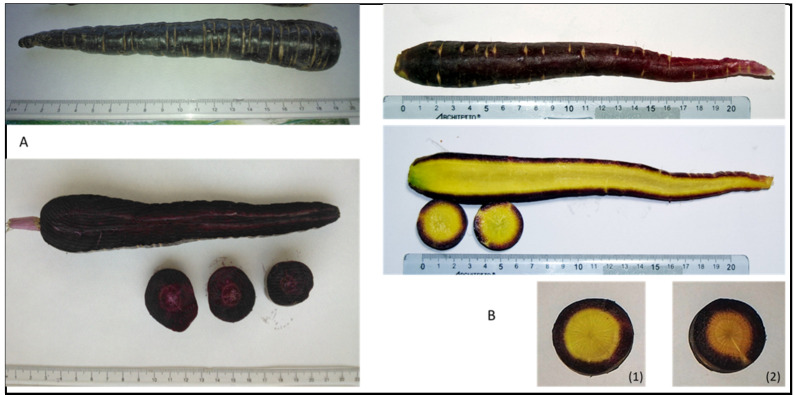
Whole and sectioned carrots (longitudinal and cross-sections). (**A**) Black carrot (BC). (**B**) “Polignano” carrot (PC); cross-sections of different types of PC are also shown, with yellow core (**1**) or orange-purplish core (**2**).

Figure 1 depicts longitudinal sections of the three coloured carrots, whereas Figure 2 shows the external and sectioned characteristics of the anthocyanin-rich carrots (black carrot, BC, and purple “Polignano” carrot, PC).

In most purple-rooted species, anthocyanins are expressed in the epidermal layer of the root, but also in the cortex (phloem), and in the xylem (core) tissues, presenting different pigmentation patterns across these tissues [7].

Among the purple genotypes we evaluated, the BC was a solid purple carrot variety selected and cultivated by the “Aureli farm”, who exports its produce all over Europe; it was uniform as a phenotype, particularly in colour (inside and outside), having a uniform “black” colour. Additionally, the commercial OC (unknown cv) was uniform in root phenotype (orange colour).

Carrots of “Polignano” of different colours are produced in a specific area (municipality of Polignano, near Bari, Apulia Region). These carrots have been listed as a landrace cultivated in Apulia Region (Southern Italy) considered at risk of erosion [19]. With the aim of rescuing and valorising local landraces, the regional administration launched, years ago, a concerted action for safeguarding the local plant biodiversity, including the PC. This carrot landrace is now cultivated in a suitable area (Bari province) by local farmers (called “keeper farmers”) and in Bari University (Agronomy Department) experimental fields. They are a mixed population, being a local landrace, not selected as a stable variety, showing a range of colours, from yellow, to deep orange, to dark purple [19], with a similar phenotype as another local landrace from Apulia, the “Tiggiano” carrot [21]. The purple type (PC) of “Polignano” carrot shows a purple cortex and a predominance of yellow core-type, but also the orange-purplish core-type often occurs (Figure 2B, carrots 1 and 2).

It is important to protect local landraces not only because they are best suited for their local environments, and therefore useful under global climatic change, but also because they represent a valuable genetic pool of biodiversity that can be exploited in breeding programmes for the introgression into commercial cultivars of targeted traits [23].

### 2.1. Anthocyanins

**Figure 3:**
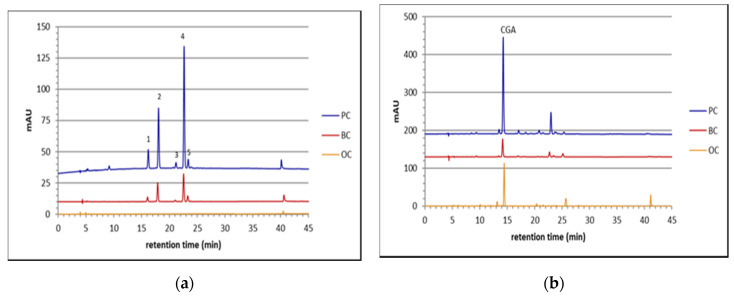
Chromatographic profiles of orange carrot (OC), BC and PC extracts at λ = 520 nm (**a**) and at λ = 320 nm (**b**). **1**: cyanidin 3-xylosyl (glucosyl) galactoside. **2**: cyanidin 3-xylosyl galactoside. **3**: sinapic acid derivative of cyanidin 3-xylosyl (glucosyl) galactoside. **4**: ferulic acid derivative of cyanidin 3-xylosyl (glucosyl) galactoside. **5**: coumaric acid derivative of cyanidin 3-xylosyl (glucosyl) galactoside. **CGA**: chlorogenic acid. BC extract was diluted by tenfold.

Anthocyanins were only detected in the BC and PC, while the OC lacked those pigments. The chromatographic profiles of the anthocyanin extracts (BC and PC) revealed the presence of five different anthocyanins, all cyanidin-based (only cyanidin was released as aglycone after acid hydrolysis; data not shown). The five peaks were tentatively identified as cyanidin 3-xylosyl (glucosyl) galactoside, cyanidin 3-xylosyl galactoside and three different acylated derivatives of the above-mentioned triglycoside (sinapoyl, feruloyl and *p*-coumaroyl) (Figure 3a).

The HPLC-DAD chromatographic conditions allowed the separation of the peaks at λ = 520 nm, but the spectral characteristics did not allow unambiguous identification of the individual peaks, apart the additional absorbance peak around λ = 320 nm, which indicates the presence of acylation with a hydroxycinnamoyl moiety. The tentative assignment to the above-mentioned compounds was done based on literature data, as many studies of black carrots reported the same anthocyanin compounds, varying only in pattern [8,26,27]. We could not find the peonidin and pelargonidin derivatives, which have been already reported, although in trace amounts [26,27,29].

As it has been reported, carrot accessions fully saturated with purple colour throughout the entire root tissues usually have the highest anthocyanin content, whereas those with purple in only the outermost tissues present low levels of anthocyanins [7].

Anthocyanins in PC and BC extracts showed some differences at quantitative levels. The total anthocyanin content (from a pool sample) in BC more than twice that in PC, on a dry weight basis (13.84 ± 0.61 vs. 5.64 ± 0.48 mg K Eq. g^−1^ DW; data not shown). The PC was very variable in anthocyanin content. As stated above, some carrots were highly pigmented in the cortex, with orange–purplish cores (carrot 2 in Figure 2B), while other carrots had purple pigmentation only in the cortex (carrot 1 in Figure 2B). The results of the anthocyanin quantification of these two different “Polignano” carrots (including BC) are reported in Table 1, specifically for different tissues (core and cortex).

BC and PC showed the same anthocyanin pattern, the feruloyl derivative of cyanidin 3-xylosyl (glucosyl) galactoside being the main anthocyanin, followed by cyanidin 3-xylosyl galactoside (Table 1). Acylation pattern is of pivotal importance for generating extended colour palettes, thereby increasing stability of the colour [30].

The anthocyanin profile of BC has been studied in detail by Smeriglio et al. [31], and it is similar to those of “Antonina” and “Deep Purple” varieties [26,27], although the anthocyanin contents reported in those studies were lower than what we found. Our results for the BC on a dry weight basis (13.84 mg. g^−1^ DW) were consistent with those reported by Kammerer et al. [8] (maximum 17 mg. g^−1^ DW) but not with those obtained by Mizgier et al. [29], who reported an exceptional total anthocyanin content (154 mg. g^−1^ DW). Other authors [32,33,34] reported different values than those we observed, but different analytical procedures were used (spectrophotometric analysis).

The major anthocyanin (the fourth one, a ferulic acid derivative of cyanidin 3-xylosyl (glucosyl) galactoside) accounted for 56% of total anthocyanins; the fifth anthocyanin (coumaroyl acid derivative of cyanidin 3-xylosyl (glucosyl) galactoside) accounted for 11%; all values were similar to the percentage peaks area reported in [26] for cv. Antonina and cv. Deep Purple.

While black carrots (BC) were uniform in phenotype and also regarding anthocyanin composition/content in the different tissues, PC showed great variation in phenotype, with a highly variable anthocyanin content/pattern. In both BC and PC, anthocyanins accumulated in the cortex particularly. It has been already reported that when both cortex and core tissues were pigmented, the total anthocyanin content in the cortex was higher than in the core [35]. Moreover, in genetic studies for anthocyanin pigmentation in carrots, three QTL regions (P1, P3 and RTPE-Q−2) have been associated with purple pigmentation of the carrot root in a tissue-specific manner [36].

In PC, we performed the extraction both from mixed PC and from individual PC, representative of the different appearance, considering separate tissues (core and cortex). In the cortex the predominant anthocyanin (anthocyanin 4) was the most variable (3.25 mg K Eq. g^−1^ DW in PC1 vs. 6.82 in PC2; Table 1). In the core the difference was less noticeable, as total anthocyanins were almost negligible in PC1, and around 0.5 mg K Eq. g^−1^ DW in PC2. As a whole, anthocyanin 4 accounted for 65% total anthocyanins in PC1, and for 84% in PC 2 (Table 1).

### 2.2. Phenolic Acids

The chromatographic characterization at λ = 320 nm allowed the identification and quantification of chlorogenic acid (CGA), the main phenolic acid present in carrots extract (Figure 3b). Other phenolic acids (caffeic and coumaric acids) were also identified, but they resulted in negligible amounts with respect to chlorogenic acid.

**Figure 4:**
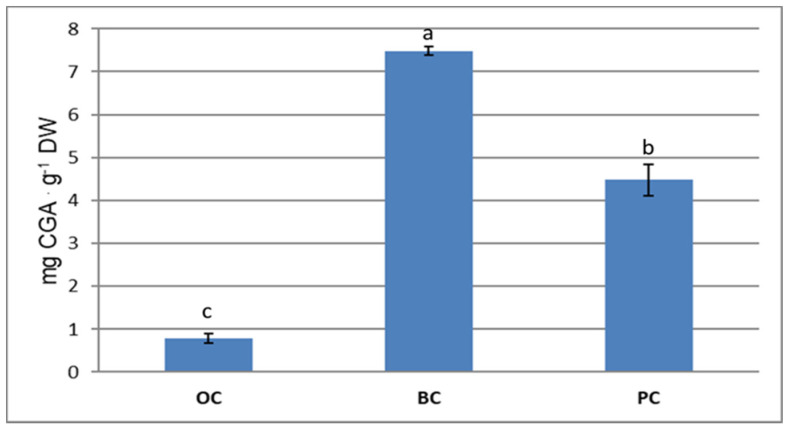
Chlorogenic acid (CGA) content (mg CGA. g^−1^ DW) in orange carrot (OC), black carrot (BC) and purple “Polignano” carrot (PC) extracts. Data are the means ± SDs (*n* = 3). Different letters indicate statistically significant differences at *p* ≤ 0.0001.

BC was the richest genotype as far as chlorogenic acid content—containing about 7.5 mg of CGA. g^−1^ DW (Figure 4).

Additionally, PC contained a considerable amount of chlorogenic acid (about 4.5 mg CGA. g^−1^ DW), while in OC the chlorogenic acid content was very low, about 0.8 mg CGA. g^−1^ DW. As for anthocyanins, phenolic acids also accumulated in the cortex at double the rate as in the core tissue (data not reported).

In BC, chlorogenic acid accounted for 30% of total phenolics (evaluated as total area at 280 nm), and in PC it made up around 40%. The total of phenolic compounds in OC was very low, with chlorogenic acid being almost 60% of the total phenols. In BC and PC there was a great contribution of anthocyanins to total phenolic content.

Other authors reported in black carrots a higher percentage of CGA among the phenolic compounds (from 57% up to 72.5%), depending on the different varieties [29,37]. In BC we found a phenolic content similar to what was reported in [8,37], while it was lower with respect to the results reported by others [29,38]. Nevertheless, the ratio between OC and BC content was confirmed. CGA’s occurrence in PC was confirmed in local purple carrots [21,39], along with the percentage of total phenolic compounds [39].

### 2.3. Hydrophilic Antioxidant Capacity

The Folin–Ciocalteu reagent (FCR) reducing capacity assay was used for the three different carrot genotypes. The FCR value for BC was statistically higher than for PC, and OC value was quite poor. Additionally, the antioxidant capacity (measured by mean of TEAC and ORAC assays) ranked BC as the highest (Table 2).

The FCR values were in accordance with the literature for OC and BC [32,34,38,40]. In particular, the FCR value obtained for BC was very similar to the one reported for cv. Antonina [26,27].

The FCR assay gives an estimation of the phenolic content of a plant extract, while it is also considered an indicator of the antioxidant (reducing) capacity of a vegetable extract, even if we perform the assays on the hydrophilic fraction (polyphenolic extract). It must be emphasized that previous studies on carrots of different colours reported a negligible and scarcely variable value for the antioxidant capacity of the lipophilic fraction, with no correlation with the total antioxidant capacity [38]. Conversely, the hydrophilic fraction was highly variable, depending on the colour [21,38].

We tested the antioxidant capacities of the three differently coloured carrots by the most reliable in vitro antioxidant assays, the TEAC and the ORAC assays [41]. The antioxidant capacity of BC was the highest and was 1.4 times and 12 times higher than those for PC and OC, respectively, by TEAC assay. As for ORAC assay, BC had a value 1.6 and 7.6 greater than those of PC and OC, respectively. Nicolle et al. [40] reported ORAC values for several coloured carrot genotypes, but they are not comparable with our results, as the assay was based on the fluorescence decay of β-phycoerythrin. OC antioxidant capacity by ORAC was consistent with the reported value [42]. Again, PC antioxidant capacity by TEAC assay was comparable to the local landrace “Tiggiano” carrot [21].

The two assays (TEAC and ORAC) rely on different antioxidant mechanisms; therefore, the contributions of different compounds (anthocyanins, phenolic acids, carotenoids) take into account this issue with resulting different values of Trolox equivalents. Overall, the antioxidant capacity of BC (and PC too, even if to a lesser extent) makes these two carrot types very interesting as food with excellent antioxidant value, similar to broccoli, purple onion, spinach and green peppers [43].

### 2.4. Carotenoids

Carotenoids in carrots are responsible for the yellow, orange and red pigmentation [2]. They represent an important portion of the bioactive compounds in carrots, and have been studied since years for their health benefits, including protection from certain cancers and cardiovascular diseases [3].

Carotenoids were analysed and quantified in the three coloured carrots (Table 3).

The total carotenoid level was statistically different in the three coloured carrots, as were the levels of single compounds, with the exception of β-carotene content, which was similar in BC and PC. OC ranked highest, except for the lutein content, which was maximized in BC, followed by PC. This is of particular interest, as lutein, although it has no provitamin A activity, has been found to be involved in the protection from age-related macular degeneration [3].

Total carotenoid contents in OC and BC were found to be consistent with the reported data on orange and purple or black carrots [38,40,44]. Those authors also reported a lutein content in purple–yellow carrots higher than the one in orange carrots, as we found. Alpha and β-carotene contents were reported instead tenfold lower in purple–yellow carrots, compared to orange carrots, as we found in BC and OC. Contradictory results were also reported, with twofold higher α and β-carotene contents or similar in purple carrots compared to orange carrots [37,44], although total carrot carotenoid content might greatly vary, depending on environmental factors and genetic factors.

Carotenoids in PC showed an intermediate content between OC (low lutein, high α and β-carotene) and BC (high lutein, low α and β-carotene). Our results are also consistent with the lutein and β-carotene content reported in another local landrace, “Tiggiano” carrot [21].

**Figure 5:**
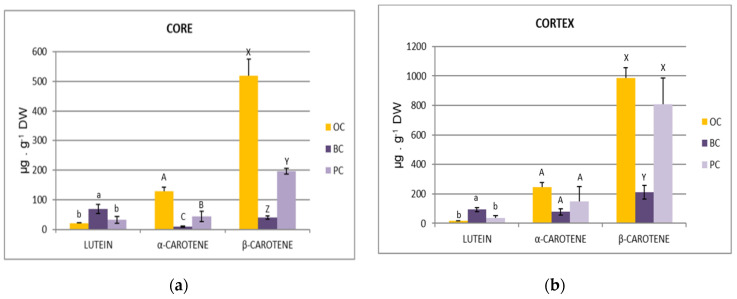
Carotenoid (lutein, α-carotene and β-carotene) contents (µg. g^−1^ DW) in the core tissue (**a**) and cortex tissue (**b**) of differently coloured carrots (OC, BC and PC). Data are the means ± SDs (*n* = 3). Different letters for each compound group indicate statistically significant differences at *p* ≤ 0.05.

Carotenoid extraction and analysis were also done for separated carrot tissues (core and cortex) (Figure 5). The results for all three coloured carrots showed that the cortex was a much richer tissue for individual carotenoids, particularly β-carotene, as has already been reported [45].

The OC cortex was rich in α and β-carotene, instead BC cortex was notable for lutein content, PC was an intermediate between OC and BC. It is worth noting that, as expected, PC showed a high variability in carotenoid content, particularly in cortex tissue, as already mentioned for anthocyanins, since PC was not a selected variety. Some carrots showed a yellow core and purple cortex, some others an orange-yellow core, with purplish shade, and purple cortex (Figure 2(B1,B2)). When analysed individually, cortex of PC2 showed a tenfold increase for α and β-carotene, with respect to PC1, with a value almost the same as OC content (Table 4).

As for anthocyanins, the differential accumulation in a tissue-specific manner was thought to be caused by the different expression patterns of genes controlling the biosynthesis of carotenoids in specific tissues [46].

### 2.5. Organic Acids and Sugars

**Figure 6:**
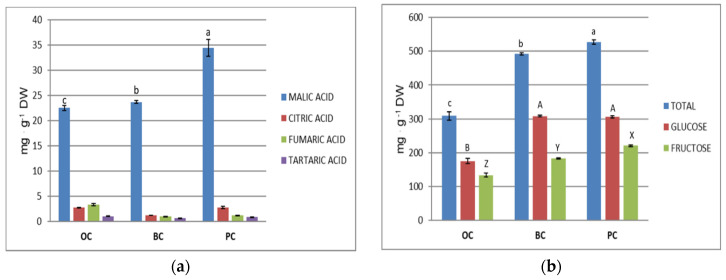
(**a**) Organic acid content (mg. g^−1^ DW) and (**b**) glucose and fructose content (and total= glucose + fructose) (mg. g^−1^ DW) in orange carrot (OC), black carrot (BC) and purple “Polignano” carrot (PC) extracts. Data are the means ± SDs (*n* = 3). Different letters for each parameter indicate statistically significant differences at *p* ≤ 0.0001.

Organic acids were analysed and quantified in OC, BC and PC. The three genotypes showed a statistically different content for malic acid. PC resulted to contain highest level of malic acid (nearly 35 mg. g^−1^ DW) (Figure 6a). The predominant organic acid in carrot is malic acid, as it has been reported [21,47], and the content we found was consistent with those reports. The same was for the content of the other organic acids detected in carrots extracts (fumaric, tartaric and citric). Citric acid in PC was around 25 mg. g^−1^ 100 g FW, which contributed to the equilibrated sweetness/acidity ratio, important for organoleptic properties [21].

In the same extract, monosaccharides were analysed and quantified (Figure 6b). The sugar content (glucose plus fructose) was statistically higher in PC in respect to BC, and even more OC, as already reported [21,45]. In BC, glucose plus fructose content was much higher than reported in previous studies on purple carrot [8,37]. Individually, glucose was found predominant over fructose in all three carrot types, as it has also been reported [8], with no difference in BC and PC. Fructose resulted more abundant and statistically different in PC instead, as fructose content has been reported to affect its relative high sweetness, in spite of a lower total carbohydrate content [45]. As a whole, OC had less sugars (glucose plus fructose).

It must be emphasized that PC, having a higher sugars and organic acids content than other carrot types, has a better taste and crispness, and this feature makes it suitable for fresh consumption. A new trend, also encouraged by the updated nutrition guideline, is the recommendation to consume raw vegetables before or during meal. PC can be suitable for this new eating behaviour and it is therefore important rescuing and valorising this local landrace. Moreover, having a lower saccharose and higher fructose content, it can be considered at lower glycemic index [45]. Few studies are reported on PC, being a local landrace, revealing interesting sensory and nutraceutical characteristics compared to commercial orange carrots, with lower sugar content in addition to sensorial traits which make this local carrot a promising raw material for new food products [19,45]. PC is also perishable and has been recently studied with the aim to improve its storage [39].

Taken all together, the analytical data which reflect the nutraceutical features of the three coloured carrots are also reported on 100 g fresh weight basis (Table 5). It is worth noting that BC ranked higher in all parameters, except for carotenoids, which were higher in OC.

Genetic improvements with biofortification (increased bioactive content) have led to designed functional foods, with public health and consumer implications [15,48]. In carrots, this has been done in the last thirty years by traditional breeding, with the creation of high carotenoid or anthocyanin-rich carrots [6,17,18]. Moreover, mining functional genes (discovered from the omic research) will promote further breeding work on carrots, and new genome technologies (i.e., the CRISPR/Cas9 system) will serve as suitable strategies for carrot improvement [30,49]. Recently, transcription factor regulatory genes have been expressed in orange carrots, producing a purple phenotype [50,51]. Alternatively, the enhanced biosynthesis of carotenoids (α and β-carotene) in black and purple carrots to the orange carrot level could be pursued with the final goal of packaging multiple bioactive compounds in a unique carrot genotype.

## 3. Materials and Methods

### 3.1. Chemicals

All reagents were purchased from Sigma-Aldrich (St. Louis, MO, USA), except authentic standards of kuromanin (cyanidin 3-*O*-glucoside chloride) and chlorogenic acid (Extrasynthèse, Genay, France); and carotenoids standards (CaroteNature, Lupsingen, Switzerland).

### 3.2. Plant Material

Three differently coloured carrots were considered: orange carrot, later on indicated as OC; black carrot, indicated as BC; and purple carrot (“Polignano” carrot), indicated as PC (Figure 1). OC, no specific cv, was purchased in a local supermarket. BC was provided by “Aureli Mario farm” (Ortucchio, AQ, Italy). Regarding the agronomic practices, the “Aureli Mario farm” use to follow the production specification of Abruzzo Region (Italy)–Global Gap, for “integrated production”.

PC samples were provided by a local farmer that cultivates those carrots in Polignano a Mare, Southern Italy (https://tinyurl.com/4m6bw4ds accessed on 15 March 2021). In accordance with the cultivation practices, fertilization was not necessary, since local farmers apply the agronomic principle of crop rotation, thus soil fertility remaining from the previous crop is sufficient to satisfy the needs of the following crop PC. More information on the agronomic practices for PC has been already reported [22].

### 3.3. Sample Preparation

Carrots (with uniform stage and size, 1–2 kg from each colour) were “topped” and “tailed”, then thoroughly washed with tap water, reduced as a pool into small pieces, then to a fine powder with a Waring blender, in presence of liquid N_2_. Some carrots were also frozen as separated tissue: phloem (cortex) and xylem (core) tissues (Figure 1) for analysing them separately.

Frozen samples were placed into calibrated tubes and put in a lyophilizer (Freezone^®^ 2.5 model 76530, Labconco Corp., Kansas City, MO, USA) for 48 h, and then stored at −20 °C. This product was defined as the dry weight (DW) of the sample.

### 3.4. Extraction and Analysis for Identification and Quantification of Anthocyanins and Phenolic Acids

Extraction of each coloured carrot (both from whole and from core/cortex tissue) was done in triplicate from 100 mg (DW) of sample macerated with 10 mL extraction solvent (Methanol:Ethanol:Water:Formic Acid = 35:35:28:2, *v*/*v*), over-night, at 4 °C, without stirring. PC extraction was done from a mixed carrot source (mixed colour), but also from separated tissues (cortex and core) of a specific coloured carrot (Figure 2B, carrot 1 and carrot 2).

After centrifugation at 3500 *g* for 10 min at 4 °C, the supernatant was recovered and the extraction was repeated on a rotary shaker at room temperature for one hour. After centrifugation (as above), both supernatants were combined and the organic solvent evaporated in vacuo at 32 °C, using a R-205 Büchi rotavapor (Büchi Labortechnik AG, Flawil, Switzerland), then brought to a known volume with acidified water (0.5% formic acid). Extracts were filtered through a 0.45 µm nylon membrane (PTFE) (Millipore, Bedford, MA, USA), stored at −20 °C and analysed by HPLC within one week.

The identification and quantification of phenolic compounds in carrot extracts was performed using an Agilent 1260 Liquid Chromatography system (Agilent Technologies, Palo Alto, CA, USA); the chromatographic conditions and column were the same already reported in [52].

Individual polyphenolic compounds were identified by comparing their peak retention times and UV–Vis spectra with those of commercial standards, and by co-chromatography of samples spiked with the standards. The identified compounds were quantified by the external standard method using a six-point calibration curve of kuromanin chloride (cyanidin 3-*O*-glucoside chloride) and chlorogenic acid (0.5–100 mg/L). For anthocyanin quantification, the amount of each anthocyanin calculated as Kuromanin Equivalent (K Eq.) was multiplied by a molecular weight correction factor [53]. Total anthocyanins were the sum of the individual anthocyanins quantified in the extract.

### 3.5. Extraction and Analysis for Identification and Quantification of Carotenoids

Carotenoids were extracted from each coloured carrot (both from whole and from core/cortex tissue) following a methodology already reported [54]. PC extraction was done from a mixed carrot source (mixed colour), but also from separated tissues (cortex and core) of specific coloured carrot (Figure 2B, carrot 1 and carrot 2). Extraction was done in triplicate from 50 mg (DW) of sample with 5 mL *n*-hexane containing 0.05% BHT. After vortexing for 2 min, and centrifugation (3000× *g* at 2 °C for 10 min), the supernatant was recovered and the same extraction was repeated four times. The combined supernatants were evaporated under N_2_ and stored at −20 °C until analysis. Before HPLC injection, samples were re-suspended in ethyl acetate (1 mL).

Carotenoids were analysed and quantified using an Agilent 1100 Series HPLC system as described by Durante et al. [55]. Total carotenoids were the sum of lutein, α-carotene and β-carotene amounts quantified in the extract.

### 3.6. Organic Acids and Sugars Extraction and Quantification

Extraction was done in triplicate from 200 mg (DW) of sample in 10 mL water with the addition of 200 mg PVPP, for 1 h at room temperature, without stirring, following the procedure reported in Blando et al. [56]. Organic acids and sugars (monosaccharides) were simultaneously analysed using the same HPLC system as above (Agilent 1100 series) equipped with a Refractive Index Detector (RID) and a UV/Vis/DAD, with an Aminex HPX-87H column (Bio-Rad, 300 × 7.8 mm, 9 μm), at 55 °C. Solvent was 0.045 N sulphuric acid containing 6% acetonitrile, with a flow rate of 0.3 mL min^−1^. Identification and quantification of individual analytes were accomplished by comparison of retention times and UV–Vis spectra with those of authentic standards, and their calibration graphs.

### 3.7. Hydrophilic Antioxidant Capacity

The Folin–Ciocalteu reagent (FCR) reducing capacity assay, the TEAC and the ORAC assays were evaluated in carrot polyphenolic extracts, as described in Blando et al. [57], with slight improvements. In particular, the incubation time for Folin–Ciocalteu assay was increased to one hour and four different dilutions were tested on the same extract. A rapid microplate methodology, using a microplate reader (Infinite M200, Tecan Trading AG, Männedorf, Switzerland) and 96-well plates (Costar, 96-well black round bottom plate, Corning), was used. All experiments were performed in triplicate, and two independent assays were performed for each sample.

### 3.8. Statistical Analysis

Statistical analysis was carried out using GraphPad Prism, version 6.00 for Windows (GraphPad software, San Diego, CA, USA). Results were expressed as mean values ± standard deviations (SDs). Differences between samples were analysed by one-way analysis of variance (ANOVA) with Bonferroni’s multiple comparison test; a *p* value lower than 0.05 was considered statistically significant.

## 4. Conclusions

The research towards developing improved vegetables in terms of nutritional quality and visual appeal can provide a sustainable, inexpensive complement to medical and social programs for preventing human disease. On the other hand, breeding programmes specifically designed for the valorisation of local landraces are seen as a promising approach toward a sustainable agricultural system. Actions aimed at preserving local landraces for their valorisation and genetic conservation purposes can be important not only for horticultural researchers, but also for producers, marketers and consumers.

On the basis of the results we obtained, BC and PC meet these features. In fact, the BC presents outstanding nutraceutical characteristics (except for carotenoids), and the PC is worth considering for a local sustainable and quality-oriented horticulture.

## Figures and Tables

**Table 1:** Individual and total anthocyanin content (mg K Eq. g^−1^ DW) in black carrot (BC) and purple “Polignano” carrot (PC) tissues (core, cortex and whole). For the PC, two different carrot types were considered (see Figure 2B).

Sample	Anth. 1	Anth. 2	Anth. 3	Anth. 4	Anth. 5	Total
***BC core***	0.25 ± 0.007 *d*	1.14 ± 0.03 *b*	0.33 ± 0.004 *c*	5.39 ± 0.06 *a*	0.39 ± 0.03 *c*	7.49 ± 0.13 *C*
***BC cortex***	1.11 ± 0.03 *d*	5.92 ± 0.19 *b*	0.50 ± 0.03 *d*	9.66 ± 0.24 *a*	2.26 ± 0.12 *c*	19.45 ± 0.62 *A*
***BC whole***	0.97 ± 0.07 *cd*	3.28 ± 0.14 *b*	0.37 ± 0.02 *d*	7.69 ± 0.27 *a*	1.52 ± 0.11 *c*	13.84 ± 0.61 *B*
***PC1 core***	n.d.	n.d.	n.d.	0.007 ± 0.0009	n.d.	0.007 ± 0.0009 *F*
***PC1 cortex***	0.20 ± 0.002 *c*	1.02 ± 0.007 *b*	0.13 ± 0.001 *c*	3.25 ± 0.03 *a*	0.24 ± 0.002 *c*	4.85 ± 0.05 *D*
***PC1 whole***	0.39 ± 0.002 *c*	1.05 ± 0.02 *b*	0.12 ± 0.006 *e*	3.28 ± 0.12 *a*	0.21 ± 0.003 *d*	5.06 ± 0.15 *D*
***PC2 core***	0.02 ± 0.0004 *b*	0.01 ± 0.003 *c*	0.01 ± 0.0004 *c*	0.43 ± 0.005 *a*	0.006 ± 0.001 *d*	0.49± 0.01 *E*
***PC2 cortex***	0.27 ± 0.0001 *c*	0.82 ± 0.0005 *b*	0.09 ± 0 *d*	6.82 ± 0.05 *a*	0.18 ± 0.004 *c*	8.18 ± 0.58 *C*
***PC2 whole***	0.32 ± 0.01 *c*	0.68 ± 0.01 *b*	0.11 ± 0.007 *e*	6.56 ± 0.25 *a*	0.14 ± 0.002 *d*	7.82 ± 0.28 *C*

K Eq. = kuromanin (cyanidin 3-glucoside) equivalent. n.d. = not determined. **Anth. 1**: cyanidin 3-xylosyl (glucosyl) galactoside. **Anth. 2**: cyanidin 3-xylosyl galactoside. **Anth. 3**: sinapic acid derivative of cyanidin 3-xylosyl (glucosyl) galactoside. **Anth. 4**: ferulic acid derivative of cyanidin 3-xylosyl (glucosyl) galactoside. **Anth. 5**: coumaric acid derivative of cyanidin 3-xylosyl (glucosyl) galactoside. Data are the means ± SDs (*n* = 3). Different lowercase letters in the same row, or uppercase letters in the last column, indicate statistically significant differences at *p* ≤ 0.05.

**Table 2:** Folin–Ciocalteu reagent (FCR) reducing capacity and antioxidant capacity (by TEAC and ORAC assays) in orange carrot (OC), black carrot (BC) and purple “Polignano” carrot (PC) extracts.

	FCR	TEAC	ORAC
Sample	mg GAE.g^−1^ DW	μmol TE. g^−1^ DW	μmol TE. g^−1^ DW
***C***	2.25 ± 0.31 *c*	6.19 ± 0.84 *c*	21.43 ± 3.06 *c*
***BC***	16.57 ± 1.13 *a*	76.67 ± 10.6 *a*	159.93 ± 3.28 *a*
***PC***	12.98 ± 0.89 *b*	54.99 ± 5.53 *b*	101.32 ± 14.17 *b*

Data are the means ± SDs (*n* = 3). Different letters in the same column indicate statistically significant differences at *p* ≤ 0.0001.

**Table 3:** Carotenoid (lutein, α-carotene, β-carotene and total) contents in orange carrot (OC), black carrot (BC) and purple “Polignano” carrot (PC) extracts, as μg. g^−1^ dry weight (DW).

Sample	Lutein	α-Carotene	β-Carotene	Total Carotenoids
***OC***	17.2 ± 1.36 *c*	255 ± 19.30 *a*	1016.35 ± 68.5 *a*	1288.55 ± 89.16 *a*
***BC***	57.58 ± 10.60 *a*	21.87 ± 3.85 *b*	60.38 ± 10 *c*	139.83 ± 24.45 *c*
***PC***	37.42 ± 4.35 *b*	47.26 ± 5.26 *b*	247.45 ± 29 *b*	332.13 ± 38.61 *b*

Data are the means ± SDs (*n* = 3). Different letters in the same column indicate statistically significant differences at *p* ≤ 0.05.

**Table 4:** Carotenoid (lutein, α-carotene, β-carotene and total) contents in purple “Polignano” carrot (PC) extract, as mg.g^−1^ dry weight (DW). PC 1 and 2 were as in Figure 2B.

		Lutein	α-Carotene	β-Carotene	Total Carotenoids
***PC1***	**core**	27.97 ± 0.93 *b*	n.d.	4.23 ± 0.05 *d*	32.20 ± 0.99 *d*
	**cortex**	48.82 ± 1.86 *a*	37.73 ± 3.02 *b*	98.95 ± 12.37 *c*	185.50 ± 17.25 *c*
***PC2***	**core**	28.35 ± 5.37 *b*	37.52 ± 4.07 *b*	206 ± 26.36 *b*	271.87 ± 35.8 *b*
	**cortex**	13.21 ± 0.37 *c*	369.65 ± 1.48 *a*	899.49 ± 7.78 *a*	1282.35 ± 9.63 *a*

Data are the means ± SDs (*n* = 3). n.d. = not detected. Different letters in the same column indicate statistically significant differences at *p* ≤ 0.05.

**Table 5:** Anthocyanins, Folin–Ciocalteu reagent (FCR) reducing capacity, carotenoids and antioxidant capacity values (by TEAC and ORAC assays) in orange carrots (OC), black carrots (BC) and purple “Polignano” carrots (PC), per 100 g fresh weight (FW).

Sample	Anthocyanins	FCR	Carotenoids	TEAC Value	ORAC Value
	mg K Eq.100^–1^ g FW	mg GAE. 100^–1^ g FW	mg.100^–1^ g FW	μmol TE.100^–1^ g FW	μmol TE.100^–1^ g FW
***OC***	n.d.	23.11 ± 3.24 *c*	14.15 ± 0.81 *a*	63.82 ± 8.24 *c*	250.7 ± 35.81 *c*
***BC***	186.85 ± 8.13 *a*	222 ± 15.13 *a*	1.82 ± 0.22 *b*	1026.43 ± 142 *a*	2159 ± 44.35 *a*
***PC***	55.13± 23.12 *b*	111.10 ± 7.61 *b*	2.81 ± 0.76 *b*	470.21 ± 47 *b*	866.3 ± 121.12 *b*

Data are the means ± SDs (*n* = 3). Different letters in the same column indicate statistically significant differences at *p* ≤ 0.0001. n.d. = not detected.

## Data Availability

Not applicable.
